# Traditional medicinal animal use by Xhosa and Sotho communities in the Western Cape Province, South Africa

**DOI:** 10.1186/s13002-019-0311-6

**Published:** 2019-07-09

**Authors:** Willem A. Nieman, Alison J. Leslie, Anita Wilkinson

**Affiliations:** 1 Department of Conservation Ecology and Entomology, University of Stellenbosch, Matieland, Western Cape 7602 South Africa; 2 The Cape Leopard Trust, P.O. Box 31139, Tokai, Cape Town, 7966 South Africa

**Keywords:** Ethnozoology, Ethnopharmacology;Informal settlements, Species accumulation curves, Xhosa medicine, Zootherapy

## Abstract

**Background:**

The use of animals and animal-derived materials in traditional medicine constitutes an important part of the belief systems of indigenous African cultures. It is believed to be rapidly expanding in South Africa, where traditional healers are estimated to outnumber western doctors by 2000:1 in some areas, with an overall clientele consisting of 60–80% of South African citizens. Despite concerns about the impact of the trade in traditional medicine on biodiversity, there has been only limited research on this topic in South Africa.

**Methods:**

Traditional Xhosa and Sotho healers operating from impoverished, rural communities in the Boland Region of the Western Cape Province were consulted to provide a comprehensive inventory of the number and frequency of animals used and sold. Species richness estimators, diversity indices, and a relative cultural importance (RCI) index were used to highlight species of concern and assess market dynamics.

**Results:**

A total of 26 broad use categories for 12 types of animal parts or products from 71 species or morphospecies were recorded. The most commonly sold items were skin pieces, oil or fat, and bones. Results showed that leopard, chacma baboon, Cape porcupine, monitor lizard species, puff adder, African rock python, and black-backed jackal were the species most used in the traditional medicinal trade.

**Conclusions:**

This study extends existing knowledge on the trade of animals in South African healing practices and provides the first attempt in the Western Cape to quantify wildlife use for cultural traditions. The results have relevance for setting conservation priorities and may assist in effective policy development inclusive of ecological sustainability priorities, as well as cultural demands.

**Electronic supplementary material:**

The online version of this article (10.1186/s13002-019-0311-6) contains supplementary material, which is available to authorized users.

## Background

Zootherapy has existed in traditional folk pharmacopoeias throughout history [1, 2] and remains an integral component in traditional medicinal practices and other cultural applications in contemporary landscapes worldwide [3, 4], particularly in African [5, 6], Asian [7, 8], and Latin American countries [9, 10].

Similarly in South Africa, the trade of, and dependence on, natural resources as traditional medicine amongst primarily indigenous African cultures is deemed to be pervasive [5, 11, 12]. African cultures and associated traditional healers in South Africa subscribe to a resolute belief that health and welfare issues are intimately connected with supernatural forces, social relationships, and ancestral relationships [13–15]. Consequently, traditional healers are highly esteemed members of the community [16] whose consultation is often preferred to those of Western doctors. Furthermore, the relatively few per capita Western doctors available in South Africa [11] have resulted in a large proportion of the country’s population being more dependent on traditional medicine [17, 18]. Estimations by several authors [17, 19, 20] suggest that between 60 and 80% of South African citizens have at some point either purchased traditional medicine or consulted with a traditional healer [18]. This is particularly relevant to communities existing in poor, rural areas (> 50% of the South African population) [21] where little opportunity exists to consult with university-educated doctors, while traditional healers in comparison are far more accessible [14].

In the Western Cape Province (WCP), the use of animals in traditional medicine or cultural practice is largely dominated by Xhosa-speaking people [22], who constitute the largest proportion of African ethnic groups in the province (24.7%, with Sotho-speaking people representing the second highest proportion at 1.1%) [23]. Animal use in Xhosa traditional medicine was documented as early as the 1930s [24], but the practice is certainly much older, since Xhosa communities had no contact with Western doctors and associated medical procedure prior to the 19th century [25]. Similar to other indigenous African cultures in South Africa, Xhosa and Sotho healing practices place an equal or greater value on the use of animal constituents and derivatives for the curing of non-medical ailments, such as protection against bad luck and witches [17, 26]. Other ‘symbolic magical’ or ‘magico-medical’ purposes include the protection against physical and spiritual enemies and entities, love charms and aphrodisiacs, increased intelligence, acquiring wealth and prosperity, and aiding pastoral enterprises [3, 15, 18, 27].

Despite its importance to indigenous communities in South Africa being widely acknowledged [6], ethnozoological research has been largely subjected to paucity, especially compared to ethnobotanical research [2, 5, 28]. The lack of ethnozoological studies in South Africa is likely due to its small claim on the greater materia medica of indigenous cultures [2], as well as the popular association of ethnozoology with ‘spiritual’ or ‘magical’ components [3, 18, 27] and the Doctrine of Signatures [29], withdrawing credibility from zootherapeutics as a realistic scientific pursuit [6]. Despite research on ethnozoology in South Africa however being largely sporadic and subject to neglect [2], there has been a recent upsurge in available information during the past few decades originating from Kwazulu-Natal [17, 30–32], the Faraday market in Johannesburg [5, 6], and the Eastern Cape Province [15, 33]—subsequently greatly improving our understanding of the topic. A noticeable gap however still remains in the WCP amid the extensive and expanding demand for animal products for traditional uses [16, 17, 34, 35], exacerbated by the growing human population [36], migratory influxes to the province [21], and high levels of unemployment [17, 37].

The aim of this study was thus to acquire information, inventory, and document the use of animal-derived materials by diviners, herbalists, and general animal parts traders (hereafter collectively referred to as traditional healers) operating from impoverished, rural communities in the Boland Region of the WCP, South Africa. A variety of quantitative approaches were employed to specifically explore the following: (1) vertebrate species incidence, richness, and diversity and (2) the species most valued by local African communities. This will provide novel insights into the extent and dynamics of the traditional healing enterprise, as well as the demand for vertebrate taxa, thus determining conservation priorities and enabling effective policy development inclusive of both ecological sustainability priorities as well as social demands.

## Methods

### Study area

**Fig. 1:**
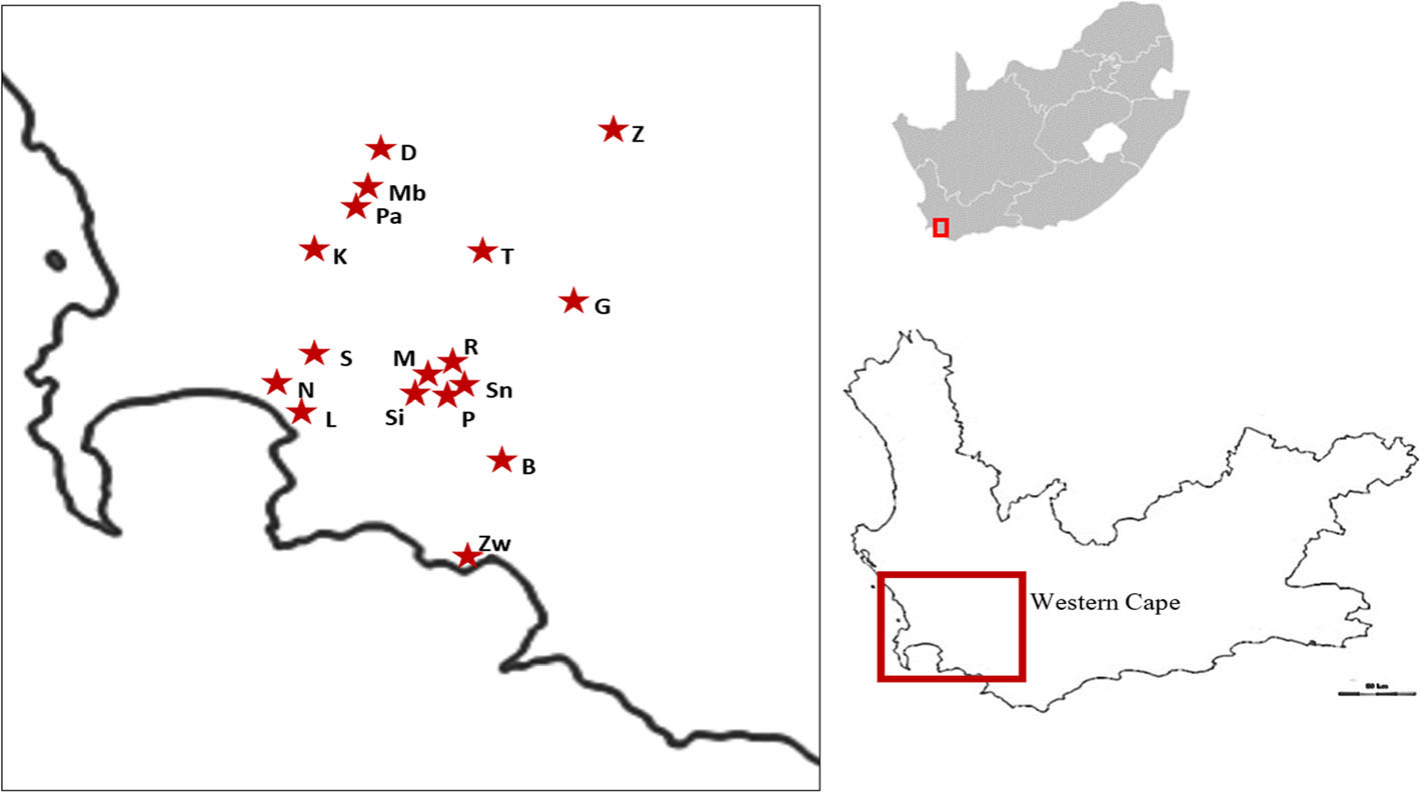
Sampled communities in the WCP of South Africa. The communities were Zweletemba (Z), Drommedaris (D), Mbekweni (Mb), Paarl SP (Pa), Kayamandi (K), Tjotjombeni (T), Goniwe Park (G), Sir Lowry’s pass village (S), Nomzamo (N), Lwandle (L), Marikana (M), Rooidakkies (R), Siyanyazela (Si), Pineview (P), Snake Park (Sn), Botrivier SP (B), and Zwelihle (Zw)

Research was undertaken in 17 townships and informal settlements in rural or peri-urban landscapes in the Boland Region (~ 4000 km^2^), part of the WCP of South Africa (Fig. 1). The sampled sites were purposively chosen to be inclusive of all such residential communities in the study area with indigenous African people contributing to > 20% of the overall population demographics. Large racial and ethnic diversity however still remained amongst sites, enabling the comparison of demographically heterogeneous and homogeneous settlements. The Boland Region consists of various protected areas enveloped by vast expansions of agriculturally transformed lands. The remaining natural habitats support low animal biomass due to the largely unpalatable and nutrient-deficient fynbos vegetation [38]. As a result, leopard (*Panthera pardus*), Cape mountain zebra (*Equus zebra zebra*), and bontebok (*Damaliscus pygargus*) are the only large mammal species that are able to persist without human intervention [38]. The WCP however boasts high levels of endemism of mammal, amphibian, reptilian, and avifauna taxa (11–54% endemism) and supports approximately 50% of all terrestrial vertebrate species found in South Africa [39]. Data collection took place in the autumn (April to May) of 2018.

### Data collection

To gather information on the use of vertebrate species by traditional healers, semi-structured interviews were conducted with 36 respondents in townships and informal settlements (*n* = 17). Unlike the more obvious and prominent *umuthi* markets found in Johannesburg and Durban [5, 6, 11, 19, 40], traditional healing in the WCP is much more discreet [22]. Consequently, identifying potential informants was achieved using a non-probability snowball sampling approach, i.e. community members were asked to locate neighbours fitting the criteria. Snowball sampling is an efficient manner of gathering information during purposive sampling in situations where there is no obvious information on the whereabouts of the population of interest [41, 42]. All interviews were conducted in the home languages of informants, either isiXhosa (81%) or Sesotho (19%). Respondents ranged from 25 to 62 years of age (median = 37.5 years) and comprised mostly males (78%). All respondents reportedly migrated from the Eastern Cape Province. Interviews ranged between 50 min and 2 h. The WCP hosts a great variety of species lineages, as well as many cryptic species (i.e. species that are hard to discern morphologically) [43]. Animal identification cards were therefore used to facilitate memory recall and to eliminate the possibility of disparity in species names or the grouping of species as ethnospecies (i.e. a folk or common name liberally assigned to a number of closely related species) [44]. Species parts or products were identified on site where possible.

All interviews were conducted anonymously, and information was kept confidential. At least a certain degree of reluctance was expected in providing information on the use of animal species, especially those relating to the treatment of magico-medicinal ailments, as previously reported [6, 17, 28]. However, respondents were almost exclusively extremely forthcoming, inviting, and even excited to have their knowledge formally documented. Only one respondent refused to participate due to recent run-ins with law enforcement (non-response = 2.7%), and uses for four species were not recorded (5.6%). Consent was obtained from each respondent for the use of their accounts prior to every interview, and ethical clearance was obtained from the Stellenbosch University ethics committee: humanities (reference: CEE-2018-6251) preceding the data collection phase of this study.

### Statistical analysis

Basic descriptive statistics were used to analyse the highest incidence of vertebrate species sold by traditional healers, as well as species sold that were of conservation concern. Species with the most uses as cited by traditional healers, the most prevalent animal parts or products, and their corresponding market values were also described.

Sampling performance was evaluated by constructing rarefaction curves, where sampling size was deemed sufficient when the expected number of species, E(Sn), did not increase with the addition of more individuals to the sample [45]. This is indicated by the ‘levelling-off’ of E(Sn) on the rarefaction curve.

Information regarding the number and frequency of species occurrence in the area [45, 46] was used to quantify the richness, diversity, and evenness or equitability of animals used and sold by traditional healers in the Boland Region. Observed species richness (S) may however be heavily dependent on sampling effort. Therefore, species richness estimation curves were used to measure and compare various estimators of species richness by adding unseen species to the observed S [47, 48], facilitating improved interpretation of S outcomes, especially since samples varied in size [46]. The performance of six non-parametric S estimators appropriate for incidence-based data (i.e. information on species frequencies), namely Chao 2, first-order jackknife (Jack 1), second-order jackknife (Jack 2), incidence-based coverage estimator (ICE), bootstrap (Boot), and Michaelis-Menten means (MMMeans), was calculated and compared. For each of the calculations, the sample order was randomised 100 times to compute mean statistics at each sample order, thereby generating smooth accumulation curves [47, 49]. Species diversity was expressed using the Shannon-Wiener index (H’), the Simpson diversity index (*λ*), Hill’s diversity numbers (N_1_ and N_2_), and Fisher’s alpha (α). Additionally, evenness was expressed with the Shannon index of evenness (J’) and Hill’s E5 [50].

All values for indices and species-richness estimators were calculated using EstimateS software v9.1.0.

The informant consensus factor (F_IC_) [51–53] was used to calculate the degree of socio-cultural coherence regarding animals being used within and amongst certain communities with respect to similar ailments. The method rests on the assumption that the greater the degree of group consensus regarding the use of ethnomedicinal species for treating certain conditions are, the greater the probability that the specific treatment is physiologically active or effective [51]. The formula in [52] was used: F_IC_ = (N_ur_ − n_t_)/(n_ur_ − 1), where N_ur_ equals the number of use citations in each use category and n_t_ equals the number of species used per use category. High values (close to 1) relate to a greater degree of informant consensus or homogeny on which animals are considered effective in the treating of a certain ailment. Conversely, low values (close to 0) indicate a high degree of variation in the number of different animals used to treat a particular ailment. F_IC_ values were only calculated for use categories with > 4 independent citations.

## Results

### Species incidence and use prevalence

The 71 vertebrate species or morphospecies cited by traditional healers in the sampled communities belonged to four classes and 20 orders (see Additional file 1). The main orders were Carnivora (20 spp.), Artiodactyla (17 spp.), and Squamata (10 spp.).

A total of 7 species and morphospecies were enumerated in > 50% of sampled communities (*n* = 17, Table 1). These were chacma baboon (*Papio ursinus*, 82.4%), leopard (*P. pardus*, 82.4%), Cape porcupine (*Hystrix africaeaustralis*, 76.5%), puff adder (*Bitis arietans*, 76.5%), genet spp. (*Genetta* spp., 58.8%), black-backed jackal (*Canis mesomelas*, 52.9%), and monitor lizard spp. (*Varanus* spp., 52.9%). Of all vertebrate animal species listed, mammal taxa (73.2%) were far more prevalent than either reptile (18.3%) or bird (7.0%) taxa. Only one vertebrate fish species was recorded. No amphibian or invertebrate (marine or terrestrial) species were identified.

**Table 1:** High incidence species in sampled communities (*n* = 17)

Mammals		Reptiles		Birds	
Common name	Samples (> 30%)	Common name	Samples (> 10%)	Common name	Samples (> 10%)
Chacma baboon	82.4	Puff adder	76.5	Owl spp.^a^	41.2
Leopard	82.4	Monitor lizard spp.^a^	52.9	Vulture spp.^a^	35.3
Cape porcupine	76.5	African rock python	41.2	Ostrich	17.7
Genet spp.^a^	58.8	Nile crocodile	29.4	Swallow spp.^a^	11.8
Black-backed jackal	52.9	Cape cobra	29.4	Cattle egret	11.8
Honey badger	47.1	Mamba spp.^a^	23.4		
Hare spp.^a^	47.1	Snake spp.^a^	17.7		
Cape clawless otter	41.2	Angulate tortoise	17.7		
African buffalo	41.2	Rinkhals	11.8		
Cape fox	35.3	Southern rock agama	11.8		
Spiral-horned antelope spp.^a^	35.3	Girdled lizard spp.^a^	11.8		
Caracal	35.3				

^a^Individuals not identifiable up to species level

Twelve species recorded (Table 2) are listed of conservation concern by the IUCN Red List of threatened species (version 2017-3). Critically endangered (CR) and endangered (EN) species included several vulture species of the genus *Gyps*, tiger (*Panthera tigris*), and an unidentified rhinoceros species (either of the genus *Diceros* or *Ceratotherium*). Additionally, several *Cordylus* spp. (girdled lizard spp.) are near-threatened (NT). African striped weasel (*Poecilogale albinucha*) and Southern African hedgehog (*Atelerix frontalis*) are listed as near-threatened in the Endangered Wildlife Trust Mammal Red List. Nile crocodile (*Crocodylus niloticus*) and Eastern green mamba (*Dendroaspis angusticeps*) are listed as vulnerable (VU) in the SANBI Red List for reptiles [54].

**Table 2:** Vertebrate species of conservation concern according to the IUCN Red List of Threatened Species (2001 categories, version 2017-3, global assessment) that were sold by traditional healers in the sampled market

Species	Common name	IUCN category	Population trend
*Acinonyx jubatus*	Cheetah	VU	Decreasing
*Aonyx capensis*	Cape clawless otter	NT	Decreasing
*Equus zebra zebra*	Cape mountain zebra	VU	Unknown
*Gyps* spp.^a^	Cape vulture	EN/CR	Decreasing
*Hippopotamus amphibius*	Hippopotamus	VU	Stable
*Hyaena brunnea*	Brown hyena	NT	Unknown
*Loxodonta africana*	African elephant	VU	Increasing
*Panthera leo*	Lion	VU	Decreasing
*Panthera pardus*	Leopard	VU	Decreasing
*Panthera tigris* ^b^	Tiger	EN	Decreasing
*Pelea capreolus*	Grey rhebok	NT	Decreasing
*Diceros/Ceratotherium* ^c^	Rhinoceros spp.	CR	Increasing

^a^Conservation status varies between species

^b^Exotic species

^c^Species unknown

**Fig. 2:**
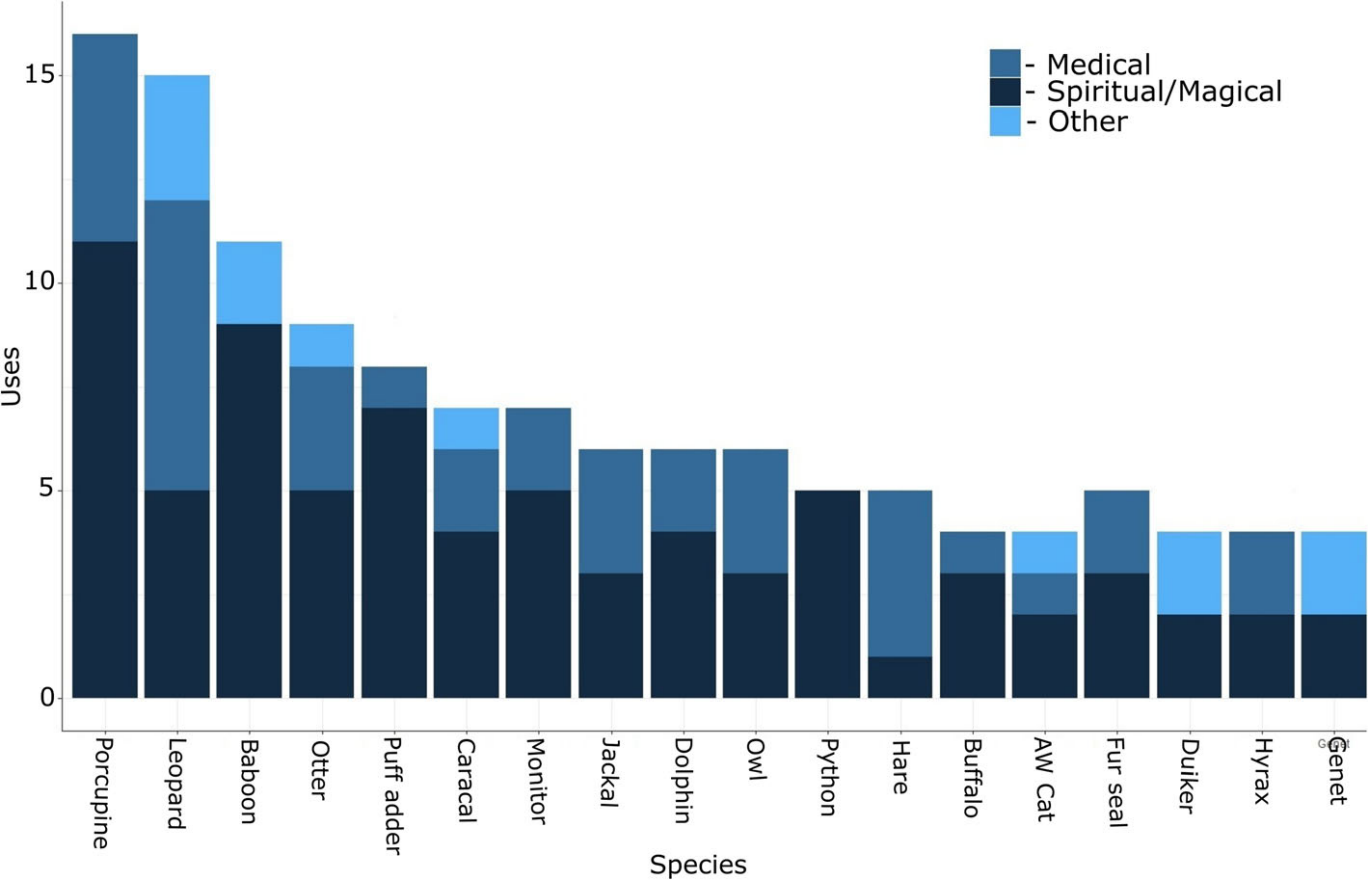
The animal species with the most use categories attributed to them by traditional healers. Bars were divided to display uses relating to medical ailments, spiritual or magical purposes, and other uses (clothing, jewellery, status symbols, etc.)

The number of uses for each animal varied from one to 16 (median = 2; Fig. 2), with the most uses attributed to Cape porcupine (16 uses), leopard (15 uses), and chacma baboon (11 uses). Uses were not recorded for four species, namely Southern African hedgehog, whale spp. (Cetaceae), tiger, and girdled lizard spp.

**Fig. 3:**
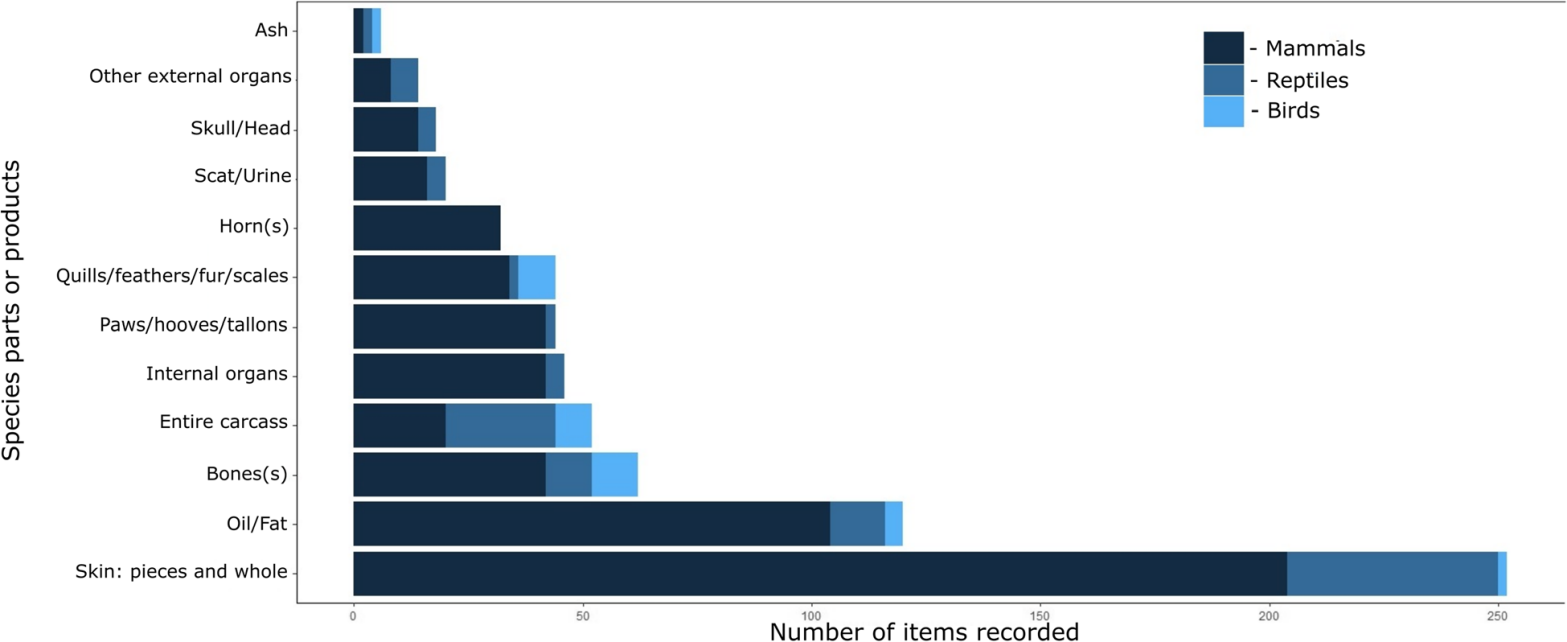
Species parts and products most frequently sold by traditional healers in the sampled community. Bars were divided to present proportional incidence of mammal, reptile, and bird taxa

A total of 716 vertebrate species parts or products were listed as being used in traditional medicinal practices in sampled communities (median = 23, range = 7–34). These were subsequently grouped into 12 categories for analyses (Fig. 3). Skin pieces and entire skins were the most prevalent form of animal constituents used or sold by traditional healers (258 items), followed by animal oil and subcutaneous fat (120 items). Animal bones (62 items), entire carcasses (56 items), and internal organs (46 items) were also highly prevalent, as well as assorted hooves, paws, and talons (44 items) and quills, feathers, fur, and scales (44 items).

High market values were recorded for wildlife items sold by traditional healers in the Boland. Prices were however highly variable across items as well as amongst the same items sold by different healers, even for the same purpose (range: USD 1–1566). The most expensive animal items were entire skins of leopard (median = USD 881, range = USD 392–1566), bones of African buffalo (*Syncerus caffer*, median = USD 646, range = USD 627–665), and bones of Cape fur seals (*Arctocephalus pusillus*, median = USD 509, range = USD 392–627).

### Sampling performance

**Fig. 4:**
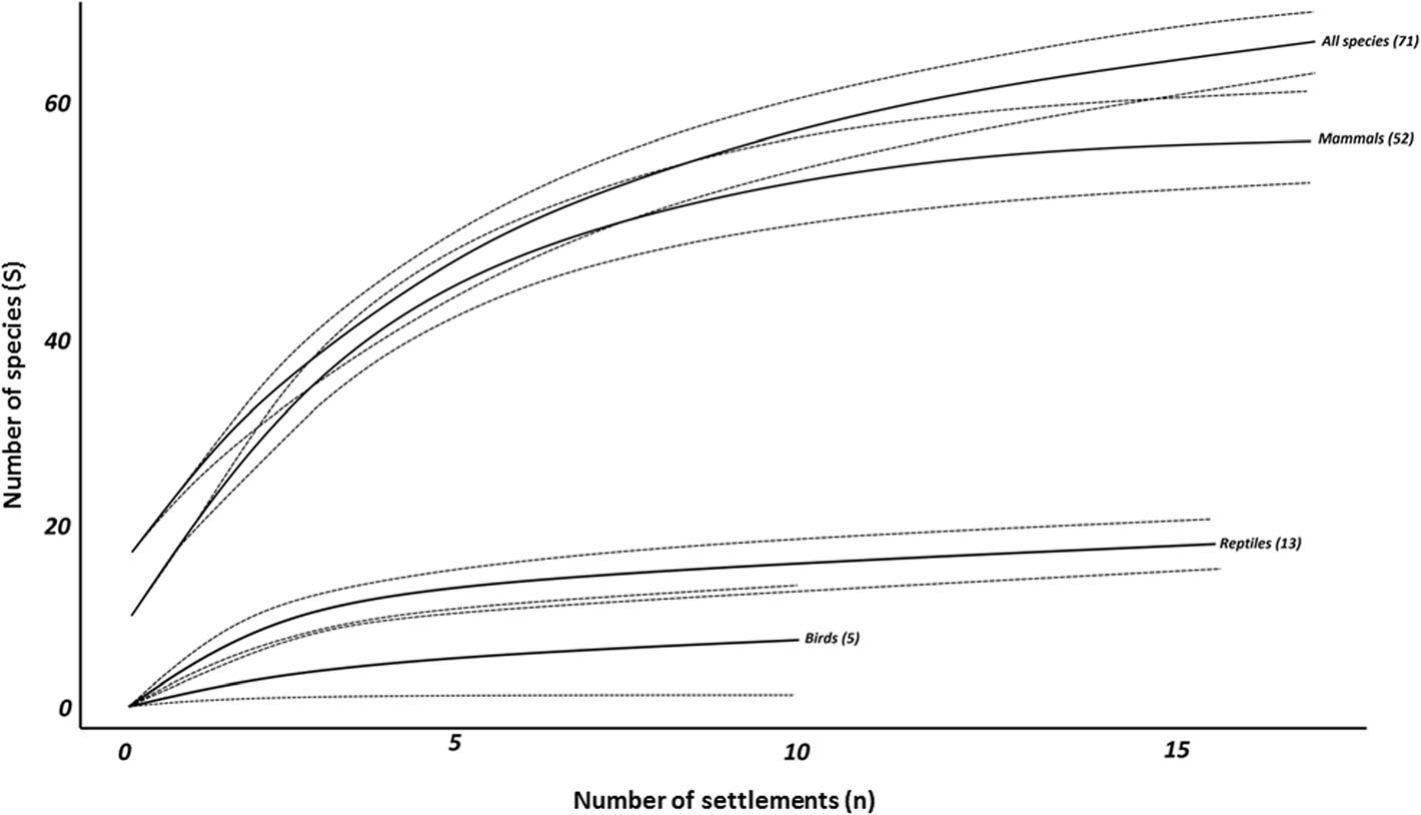
Rarefaction curves (solid lines) for vertebrate animals used by traditional healers in informal settlements in the Boland Region (*n* = 17), with 95% confidence intervals (dashed lines). Parentheses indicate sample sizes

Sample-based rarefaction curves were plotted for vertebrate animal taxa sold by traditional healers in the Boland Region, along with corresponding 95% confidence intervals (Fig. 4). None of the samples truly reached an asymptote. However, they were all clearly approaching an asymptote as the rate at which new species were found gradually decreased. Therefore, the sampling size was said to be sufficient [11, 55]. Further sampling of traditional healers would thus not yield a significant number of novel, undiscovered species for any of the individual vertebrate classes. The rapid initial accumulation shown by reptiles and bird groups indicated that species were accumulating faster for comparatively small sample sizes. Species richness was correlated with the number of settlements in which the vertebrate class was found.

### Species richness, diversity, and evenness

Species richness estimates and other summary values were calculated for three sets of data: the first containing information on all species recorded in the sampled communities, the second only mammal species, and the third only reptile species (Table 3). Bird taxa were excluded from analysis due to their relatively low incidence in the sampled community.

**Table 3:** Species richness estimates and other values characterising the use of animals by traditional healers in the WCP

	All species	Mammals	Reptiles
Samples (*n*)	17	17	16
Individuals (*N*)	450	365	67
Observed species richness	71	52	13
Estimated species richness			
ICE	74.7	54.5	13.6
Chao 2	73.3 ± 3.0	55.4 ± 3.2	13.0 ± 1.8
Jack 1	79.4 ± 2.1	58.5 ± 1.5	14.9 ± 0.8
Jack 2	78.3	61.5	14.2
Bootstrap	75.5	55.8	14.2
MMMeans	85.8	61.1	14.5
Singletons^1^	9	7	1
Doubletons^2^	13	6	3

Bird taxa were excluded due to their relatively low incidence in the market

^1^Number of species occurring only once across all samples

^2^Number of species occurring only twice across all samples

**Fig. 5:**
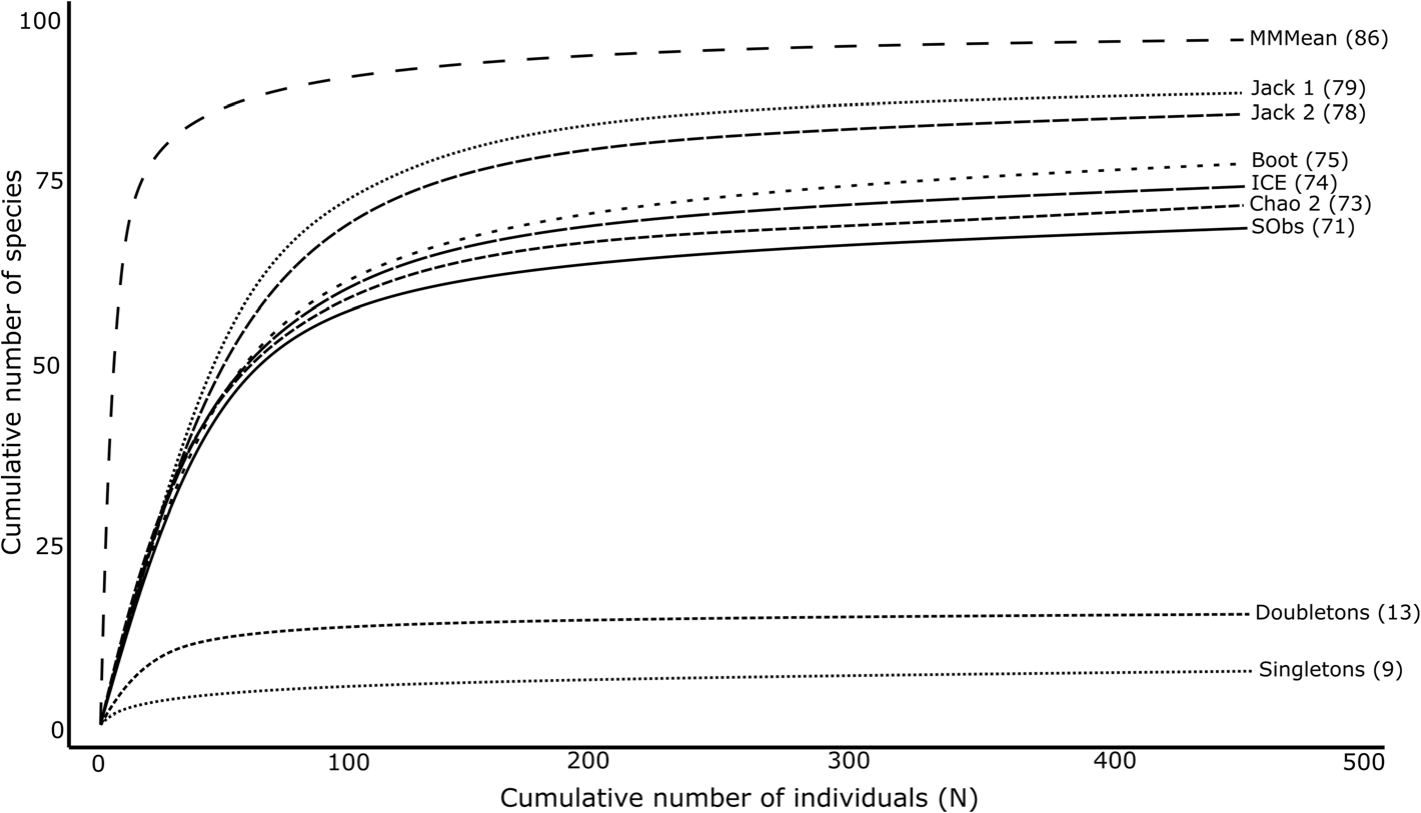
The comparative performance of six incidence-based species richness estimators (MMMeans, ICE, Jack 1, Jack 2, Bootstrap, and Chao 2), as well as the observed species accumulation curve, for all vertebrate species recorded (*n* = 71). The cumulative numbers of singletons and doubletons were also plotted. Estimated species richness is indicated in brackets. Samples were randomised 100 times

All estimators appeared to approach an asymptote sooner than the observed species accumulation curve (Fig. 5), indicating their usefulness as adequate species richness estimators [56]. The MMMeans estimator understandably reached an asymptote first, given its asymptotic nature [57]. The remaining estimators approached asymptotes in relative parallel to the observed species curve. The difference between the highest estimator (MMMeans) and lowest estimator (Chao 2) was 12.5 species. The observed species richness was 83% of the highest richness estimator. Plots of singletons and doubletons rose rapidly at the first samples and then levelled-off as sample size increased.

The overall species diversity recorded (Table 4) was relatively high (Shannon H’ = 3.79; Simpson’s 1/λ = 28.55). The high diversity values relate to high levels of uncertainty, i.e. it would be difficult to correctly predict the identification of the next species found if sampling were to continue. Amongst taxonomic groups, mammals had comparatively higher diversity values (Shannon H’ = 3.47; Simpson’s 1/λ = 20.96) than reptiles (Shannon H’ = 2.02; Simpson’s 1/λ = 7.59) and birds (Shannon H’ = 1.47; Simpson’s 1/λ = 3.92), which was correlated with species richness between these groups. The low value for Fisher’s alpha was expected due to the small number of species recorded compared to other ethnoecological studies [5], especially ethnobotanical surveys [11, 46].

**Table 4:** Selected measures of diversity calculated for vertebrate taxa (together and separately) sold by traditional healers in informal settlements in the Boland Region

Index/measure	Animals (*n* = 17)	Mammals (*n* = 17)	Reptiles (*n* = 16)	Birds (*n* = 10)
Individuals	450	365	67	17
Species richness (S or N_0_)	71	52	13	5
Mean S per community ± SD	18.2 ± 5.7	14.4 ± 5.0	4.12 ± 1.76	1.29 ± 1.16
Shannon-Wiener index (H’)	3.79	3.47	2.02	1.47
Simpson’s (1/λ) = Hill’s N_2_	28.55	20.96	7.32	3.92
Hill’s N1 (e^H’^)	44.26	32.37	7.54	4.35
Fisher’s α	23.22	16.12	4.25	2.13
E1 (Shannon J’) = (H’/H’_max_)^a^	0.89	0.88	0.79	0.91
Evenness E5 (N2-1/N1-1)	0.64	0.64	0.97	0.87
Mean number shared species	6.58 ± 3.28 (range 1–14)	5.28 ± 2.85 (range 1–13)	1.40 ± 1.02 (range 0–4)	0.22 ± 0.45 (range 0–2)

^a^H’_max_ = ln(S)

Shannon J’ evenness values were high overall (range = 0.79–0.91), indicating that most species were evenly dispersed throughout the sampled communities and that relatively few species were dominant. The low value for reptiles was likely due to the dominance of puff adder, monitor lizard spp., and African rock python. By comparison, E5 evenness values for all species and mammals were smaller (0.64 each), potentially as a result of the dominance of leopard and baboon in the samples. Overall, the high evenness values indicated that traditional healers in the Boland Region did not necessarily narrowly favour a few specific animal species, even though some species were more popular than others. Evenness values should be interpreted with caution since a scarcity of popular species during the time of sampling may have led to species being underreported.

### Informant consensus factor

The F_ic_ analysis identified definite variations in the degree of heterogeneity and homogeneity in the selection of different animals to treat certain conditions by traditional healers operating in informal communities (Table 5). The highest degree of informant consensus (i.e. high socio-cultural coherence) was for resolving court cases or reducing prison sentences (F_ic_ = 0.88) and for treating mental illnesses or improving cognitive abilities (F_ic_ = 0.85). Informants thus strongly agreed that black-backed jackal and Cape porcupine were superior animals for resolving court cases or reducing prison sentences, while mental illness or cognitive vigour was best treated with black-backed jackal, Cape fox (*Vulpes chama*), bat-eared fox (*Otocyon megalotis*), and leopard. The lowest degree of informant consensus (F_ic_ = 0.00) was for treatments of cold or flu, headaches, kidney stones, and shingles, as well as for love charms and for protection against crime. All species mentioned by traditional healers for the treatment of these ailments were different from each other. Respondents exhibited comparatively lower consensus in the treatment of medical ailments (AverageF_ic_ = 0.31) compared to the treatment of spiritual or magical ailments (AverageF_ic_ = 0.36). Overall consensus for treating any ailment was however low (AverageF_ic_ = 0.34).

**Table 5:** Socio-cultural coherence in the treatment of commonly cited medical ailments within and between sampled communities measured with the informant consensus factor (F_ic_)

Ailment	N_t_	N_ur_	F_ic_
Resolve court cases/reduce prison sentence (SM)	2	9	0.88
Mental illness treatment/improved cognitive ability (M)	4	21	0.85
Improved muscle development/physical appearance (M)	5	14	0.69
Stroke (M)	3	7	0.67
Protect against black magic/bad muti/idliso^1^/harm (SM)	6	12	0.55
Rituals/summon spirits (SM)	5	8	0.50
Spiritual protection: infants (SM)	3	5	0.50
Spiritual protection: individual (SM)	33	62	0.48
Spiritual protection: homestead (SM)	15	26	0.44
Predict future/enlightenment (SM)	5	8	0.43
Acquire wealth or good fortune (SM)	10	16	0.40
Fertility (M)	4	6	0.40
Protect livestock/crops (SM)	4	6	0.40
Epilepsy (M)	8	12	0.36
Elicit black magic/harm/idliso^1^ to others (SM)	11	16	0.33
Ibekelo^2^ (SM)	5	7	0.33
Increased agricultural yield (SM)	5	7	0.33
Paralysis (M)	4	5	0.25
Exorcism (SM)	9	11	0.20
Arthritis (M)	7	8	0.14
Cold/flu (M)	4	4	0.00
Headaches (M)	4	4	0.00
Kidney stones (M)	4	4	0.00
Shingles (M)	5	5	0.00
Love charms (SM)	4	4	0.00
Protect against crime (SM)	4	4	0.00
Average	6.65	11.19	0.34

*N*_*t*_ number of species per use category, *N*_*ur*_ number of citations per use category. F_ic_ values were only calculated for use categories with > 3 citations. Ailments: *M* medicinal, *SM* spiritual/magical

^1^Idliso (translates to ‘African poison’) refers to a cultural phenomenon in which black magic is used to poison the food of others, with the intention to induce serious illness, misfortune, or death

^2^Ibekelo is a form of black magic poison that is placed on the walking path of the victim. It is believed that when the victim steps over it, he/she will become seriously ill. Illness is said to begin in the limbs, gradually moving upward

## Discussion

The use of vertebrate species in traditional medicine is a controversial subject that has raised many concerns regarding its impact on natural wildlife [33, 58], and several statements have been made on the widespread abundance of traditional healers in South Africa [6, 11, 59]. This study not only substantiates those claims, but emphasises the previously undocumented relevance of the traditional medicinal trade in household businesses in the Boland Region of the WCP.

This study recorded 71 vertebrate species and morphospecies being used in traditional medicinal practices, constituting *c*. 4.5% of all mammal, reptile, and bird fauna found in South Africa (1 488+ species, not including marine mammals). In accordance with previous South African ethnozoological inventories [5, 17, 32, 33], mammals were the most prominent taxonomic group (73%, 11 orders). Contrastingly, studies in Latin America have found traditional medicinal practices to place an equal or greater value on invertebrate, reptile, and bird taxa compared to mammal taxa [58, 60, 61]. The current study however recorded reptiles (18%, three orders) and birds (7%, five orders) to a far lesser extent. Additionally, a single shark morphospecies was recorded, and no amphibian or invertebrate (marine or terrestrial) species. Domestic animals are generally viewed as an unimportant source of medicine [5], and consequently, only three domestic species were recorded. The species most cited during ethnoecological inventories may however provide a false representation of the actual species abundance in the markets, especially when sampling only occurs in one season [62].

### Sampling performance and completeness of the dataset

To evaluate sampling performance and completeness of the dataset, rarefaction curves were constructed, as well as other species accumulation curves based on a variety of species richness estimators. The rarefaction curves never truly reached an asymptote, as required to deduce sufficient sampling effort [48]. However, given the large area from which animals can be sourced, the relatively large study area, and the diversity of species sold in traditional medicine markets [46], it is unlikely that a true asymptote will ever be reached [11]. Therefore, species richness estimators predicted that a number of species were not recorded in the study area, especially mammals, which had a greater number of singletons present [11]. Based on the rate at which estimators reached an asymptote and their consensus with each other and other estimators [56], the Jack 1, Jack 2, and MMMeans estimators were considered the best estimators. The MMMeans estimator predicted that 86 species could be identified by further surveying in the sampled communities (i.e. 15 new species), while the Jack 2 and Jack 1 estimators predicted that a further seven and eight species could be found, respectively (Fig. 5). Based on these estimators, the current study thus recorded 83–91% of the possible species in traditional medicine in the Boland Region’s communities, which is more than adequate [55]. However, since many species were classified as morphospecies where no distinction was made between species by traditional healers, species richness is likely to be higher than reported in this study. Furthermore, since data collection occurred over a relatively short time period, some homogeneity is expected in the species recorded, and more species could therefore likely be found in subsequent studies spanning different seasons. Nevertheless, the species accumulation curves were all approaching asymptotes or had levelled-off, from which can be concluded, in conjunction with the rarefaction curves, that sampling size was sufficient and further sampling would likely not yield significantly more species.

### Species’ uses

Understanding the specific uses of species and the factors affecting medicine choices can shed light on whether or not alternative therapies can be realistically suggested [63]. In this study, specific uses attributed to individual species greatly varied between species, as well as between traditional healers within and between communities. The Cape porcupine, leopard, and chacma baboon were the most diversely utilised, with 16, 15, and 11 distinct uses being attributed to them, respectively. Concerns are therefore expressed towards the sustainability of these species by virtue of the number of uses attributed to them. Additionally, definite variations in the degree of heterogeneity and homogeneity in the selection of certain animals to treat certain conditions were observed. The ailments listed by traditional healers were grouped into 26 categories (Table 5), and the majority of these categories were found to relate to spiritual or magical ailments (58%), displaying a greater degree of socio-cultural coherence in the species used for treating these ailments as opposed to medical ailments. Overall, informant consensus for treating any ailment was however relatively low (0.34). It is unclear why these large discrepancies exist, but it undoubtedly decreases the validity of these treatments as viable solutions to medical afflictions. Beyond species inconsistencies, a plethora of animal parts and products are furthermore used for the treatment of various ailments. These include predominantly skin pieces, oils and fats, and animal bones. The low degree of socio-cultural coherence identified in this study is therefore actually much lower, and it would be of great conservation benefit to increase culturally sensitive educational programmes to promote the use of western medicine in the treatment of purely medical ailments. The majority of informants in this study were certified by traditional healer and herbalist associations, which urges them not to discourage the use of western medicine, but rather supplement its use with natural products. A dislike or antagonism towards western medicine is thus not prevalent in these communities as popularly believed. Although the demand for traditional medicine is frequently reported as growing [16, 17, 34, 35], it was evident in this study that many considered the use of traditional medicine as ‘outdated’ and ‘not part of their modern belief systems’. As observed on more than one occasion, consumers of the trade travelled substantial distances to seek the help of traditional healers due to the fear of being judged or ridiculed in their respective communities. However, the majority of listed treatments are related to spiritual ailments, especially for the protection against evil spirits and demons, and can thus not realistically be scientifically screened or tested, inhibiting the ability to prescribe alternative medicines.

### Species’ selection

It would benefit conservation and educational programmes to understand the psychology underlying the selection of species for traditional medicine. For example, the highest degree of informant consensus in this study was observed for the treatment of mental health issues and improved cognitive function through the use of jackal and fox species. The use of these species is likely founded on the general cross-cultural recognition of the cunningness of these animals and their popular portrayal as sly and clever characters in folk tales and fables. The selection of species for use in traditional medicine however depends on various anthropological, behavioural, and phenotypic factors [64], and it is not always easy to deduce the thinking pattern underlying the use of certain species. Nonetheless, morphology or ‘signatures’ have been suggested on numerous occasions as having a substantial influence on the species chosen for medicinal purposes [6, 65, 66]. This study similarly found further evidence for these trends. For example, porcupine quills were used in preparing and administering medicine because the porcupine is known to largely feed on bulbs and tubers which are often employed in ethnobotanical medicine, cattle egret (*Bubulcus ibis*) were used to guard homesteads because they are seen ‘guarding’ livestock as they characteristically walk alongside them, and rinkhals (*Hemachatus haemachatus*) were used to protect crops against hail since it is believed that the characteristic hood of the cobra can cover the crops. Furthermore, since traditional medicinal practitioners believe animal traits can be transferred from animals to people, antelopes and hares were often avoided because they were viewed as scared and weak animals, while apex predators such as leopard, lion, and crocodile are highly sought after to transfer properties of strength and dominance [6].

### Economic aspects of the trade

Identifying the prices given to products may elucidate factors relating to consumer behaviour and preferences [67, 68], as well as local purchasing power [69]. The market value of items used and sold in the sampled community was highly variable, ranging from USD 1 (baboon oil) to USD 1566 (leopard skin). Leopard products were highly popular and were consistently the highest priced. Items from Cape fur seals, dolphin spp., vulture spp., aardwolf (*Proteles cristata*), and African buffalo were also abnormally expensive, likely due to the difficulty in obtaining these items. It is however worth noting that few items could be acquired for less than USD 40, and the majority exceeded USD 80. In some instances, traditional healers explained that expensive products and treatments are acquired in dividends, primarily divided into ‘deposit’ payments and ‘finalising’ payments. Others welcomed the outmoded form of bartering, for instance releasing a product in exchange for livestock (e.g. poultry, goats or cattle) or tools (e.g. grinding stones). Interestingly, one informant explains the considerable variation in prices by stating: “…many of them [traditional healers] exploit customers to get money… for example in asking R2 000 (USD 157) for a genet skin… while others, like me, don’t care about the money, but only the acknowledgement… gratification from a higher deity is far greater than money” (translated from isiXhosa). This sheds light on the extent to which traditional healers are motivated by financial rewards, potentially also explaining why traditional healers in the sampled communities varied in their involvement in the industry, and their reliance on the income generated. For example, a number of informants had full-time jobs in a diversity of vocations, and only practiced healing on weekends, while others practiced healing full-time. There was also a great disparity in living conditions and dependency by other family members, all potentially influencing market prices. Although the motivations stated for becoming traditional healers were relatively coherent across informants (mostly through divine revelation), it is undeniable that some healers are at least partially driven by monetary requirements, fulfilled by the growing and lucrative traditional healing industry [59]. It is therefore not so farfetched to consider the possibility of ‘fake traditional healers’, intent on generating income through deceitful practice. In fact, one informant stated “we [the community] do not visit the hoaxes [fake healers], but rather travel to the Eastern Cape to see the real diviners” (translated from isiXhosa). Future policy interventions need to take these motivations into consideration and adjust their approaches to these separate groups accordingly [22]. For many traditional healers or general traders, selling traditional medicine is not their primary choice of occupation, but necessitated by a lack of broader education and skills, as well as job scarcity [70]. Therefore, a real need exists to invest in the education of these communities to equip and enable them to pursue careers beyond their current prospects.

### Species’ diversity and evenness

Overall, the species diversity in the sampled communities was relatively high, as observed in most ethnoecological studies [5, 11, 45, 46]. Since uncertainty is synonymous with diversity [71], the relatively high diversity values thus imply greater difficulty in correctly predicting the species found in a subsequent sample and indicate a low probability that two species picked at random from the sample belong to the same species [46, 50]. Within taxonomic groups, diversity was consistent with the species richness of those groups and the number of singletons recorded in those groups. Likewise, overall evenness values were relatively high, substantiating that most species were evenly dispersed throughout our sampled communities. Reptile taxa were however dominated by a few species, namely puff adder, monitor lizard spp., and African rock python, resulting in lower evenness values than other taxonomic groups. Traditional healers therefore did not narrowly favour a few specific animal species, but utilised a plethora of different species. However, despite high evenness values, it was apparent that the species sold varied between communities. The reasons for these discrepancies remain unclear; however, it is worth noting that the species sold in communities consisting of Sotho natives differed significantly from the species sold in Xhosa communities.

### Sustainability of the trade

Twelve species of conservation concern were recorded during this study (11 mammals, 1 bird), raising concerns about their continued existence alongside the rapidly expanding traditional healing market. The majority of species enumerated that were of conservation concern however occurred in small quantities, understandably considering their presumed scarcity. Leopard, lion (*Panthera leo*), Cape clawless otter (*Aonyx capensis*), vulture species, and brown hyena (*Hyaena brunnea*) were however found in high densities. Less attention is usually given to species not of immediate conservation concern. However, given the regional scarcity of many species abundant in other regions of Southern Africa (e.g. black-backed jackal, aardwolf, aardvark (*Orycteropus afer*), vulture spp., and monitor lizard spp.), the ecological sustainability of the use of these animals is also questionable. Indiscriminate harvesting of these species from likely standardised locals and protected areas, coupled with increased migration patterns into the WCP [21], threatens conservation efforts and ultimately species survival.

### Limitations and future research

This study took the first step in improving the overall understanding of the requirements and demands associated with the traditional healer-belief system, but failed to identify and map the sources providing the animal material for traditional healers. Informants were generally reluctant in revealing their sources, as reported previously [5], only admitting that material is sourced locally as far as possible, and only imported from areas such as Johannesburg, Durban, and the Eastern Cape Province when they cannot be found locally. At least one informant reported that materials are sourced exclusively from outside of the WCP, and four respondents mentioned that local farm workers supply them with animal carcasses in exchange for monetary compensation. Another informant claimed to be part of a group of traditional healers that together employ the services of a local, licensed professional hunter. It seems, therefore, that although large quantities of animal material are sourced from outside of the WCP, the market inarguably places some measure of pressure on local wildlife populations. Furthermore, this study is limited by a lack of knowledge on the actual turnover of species, since the rate at which products are sold and replaced was not obtainable. The lack of knowledge on the source populations or localities for animal harvesting, and the specific demand for products, will hinder conservation efforts and effective mitigation development, such as the identification of target areas for restoration initiatives. It would thus be of great future benefit to identify sources of animal material to incorporate these into effective policing and development frameworks that promote wildlife conservation and welfare, while still ensuring access to wildlife and wildlife uses that are deemed culturally acceptable. Respondents to the study made no mention of items being exported to other regions. However, given the global relevance of the medicinal animal trade [72], as well as the demand for African species’ products in other parts of the world [73], future research may also benefit from identifying market influences beyond the scope of the current study.

## Conclusion

Ethnozoological studies have the potential to make a valuable contribution to elucidating biodiversity demands globally, thereby guiding concomitant conservation efforts [74]. Additionally, studies of this nature effectively reveal human-related factors related to biodiversity decline and provide opportunity for involving local communities in conservation interventions. This study extends existing knowledge on the trade of whole animals and animal-derived products in South African traditional healing practices and provides the first quantification for the practices in the WCP. The species inventory provided here is comprehensive and will therefore provide valuable information to inform management decision-making. Considering all analyses employed in this study, we recommend future investigations and subsequent conservation interventions assign special attention to the following species: leopard, chacma baboon, Cape porcupine, monitor lizard spp., genet spp., puff adder, African rock python, vulture spp., and black-backed jackal. The pressures exerted on these species by traditional healing, as well as their importance in the trade, were highlighted. Of these species, those that are range-restricted, threatened, habitat-specific, or occurring in small population sizes [6] are most at risk of localised extinctions. The newly described and significant threat that traditional medicine poses to leopard populations in the WCP cannot be overly emphasised. In contrast to similar studies in other parts of South Africa [5, 33], leopards in this study had the highest incidence amongst communities (82%), had the second most uses attributed to them (15 uses), and were considered important in the treatment of various ailments. These results, coupled with the severe reduction and isolation of leopard populations during the past few decades due to habitat fragmentation, reduced prey bases, and human-wildlife conflict [75, 76], present a potential threat to their continued existence. Finally, further concern stems from the increased probability of the spread of zoonoses amongst traditional healers and the users of the trade [10, 77, 78], often residing in close proximity to one another. Policymakers, conservationists, and managers are now thus faced with an immense challenge, namely to further assess the dynamic trade of animals for traditional medicine, and subsequently generate policies and interventions that balance socio-economic development and cultural requirements with the diverse demands of biodiversity.

## Additional file

Additional file 1: **Table S1.** Complete list of recorded species and their respective uses. (DOCX 22 kb)

## Data Availability

The dataset supporting the conclusions of this article is included within the article (and its additional file).

## Abbreviations

CR: Critically endangered
EN: Endangered
F_IC_: Informant consensus factor
ICE: Incidence-based coverage estimator
MMMeans: Michaelis-Menten means
NT: Near-threatened
RCI: Relative cultural importance
S: Species richness
VU: Vulnerable
WCP: Western Cape Province
