# Supplementary material for: Traditional medicinal animal use by Xhosa and Sotho communities in the Western Cape Province, South Africa
Source: J Ethnobiol Ethnomed. 2019 Jul 9;15:34. doi: 10.1186/s13002-019-0311-6 (PMC6617652; doi:10.1186/s13002-019-0311-6)
Supplement: Supplementary file 1 — Table S1. Complete list of recorded species and their respective uses. (DOCX 22 kb) [file 13002_2019_311_MOESM1_ESM.docx]

**Additional file 1**

All vertebrate species (n = 71) documented in the sampled communities (n = 17), with corresponding parts and products utilised, the purpose or use of those animal constituents, and the market value of each component. NIA = No information available.

| **Class** | **Order** | **Genus and species** | **Common name** | **Part/product used** | **Purpose/Use** |
| --- | --- | --- | --- | --- | --- |
| Aves | Pelecaniformes | *Bubulcus ibis* | Western cattle egret | Entire carcass^1^ | Protect home from evil spirits^1^ |
| Aves | Accipitriformes | *Gyps* spp. | Vulture spp. | Skin piece^1^  Feathers^2^  Bone(s)^3^ | Protection against evil spirits^1,2^  Arthritis treatment^2,3^  Remove evil spirits from home^3^ |
| Aves | Passeriformes | *Hirundo* spp. | Swallow spp. | Entire carcass | Protection against lighting  Induce lightning to kill others  Protection against evil spirits |
| Aves | Strigiformes | *-* | Owl spp. | Oil^1^  Entire carcass^2^  Ash^3^  Feathers^4^  Subcutaneous fat^5^ | Arthritis treatment^1^  Kidney stones treatment^1^  Protection against evil spirits^2^  Remove evil spirits from home^3,4^  Stroke treatment^5^  Prevent material theft^5^ |
| Aves | Struthioniformes | *Struthio camelus* | Common ostrich | Bone(s) | Promote physical growth  Protection against evil spirits |
| Chondrichthyes | Selachimorpha | *-* | Shark sp. | Skin piece | Protection against evil spirits |
| Mammalia | Carnivora | *Acinonyx jubatus* | Cheetah | Skin piece | Transfers strength |
| Mammalia | Artiodactyla | *Antidorcas marsupialis* | Springbok | Horn^1^  Skin piece^2^ | Clothing/decoration^1,2^  Rituals^1,2^  Community status^2^ |
| Mammalia | Carnivora | *Aonyx capensis* | Cape clawless otter | Viscera^1^  Skin piece^2^  Scat^3^  Skull^4^  Entire carcass^5^ | Epilepsy treatment^1^  Clothing/decoration^2^  Protection against evil spirits^2,5^  Increase crop yield^2,3^  Cold/Flu treatment^2^  Shingles treatment^2^  Predict future^4^  Spiritual enlightenment^4^  Cause misfortune^4^ |
| Mammalia | Carnivora | *Arctocephalus pusillus* | Cape fur seal | Oil^1^  Skin piece^2^  Bones(s)^3^ | Shingles treatment^1^  Protection against evil spirits^1^  Attract good fortune^2^  Paralysis treatment/induce^3^ |
| Mammalia | Eulipotyphla | *Atelerix frontalis* | Southern African hedgehog | Quills | *NIA* |
| Mammalia | Cetacea | *-* | Whale spp. | Oil | *NIA* |
| Mammalia | Carnivora | *Canis mesomelas* | Black-backed jackal | Subcutaneous fat^1^  Fur^2^  Skin piece^3^  Bone(s)^4^  Liver^5^  Oil^6^  Brain^7^  Urine^8^ | Reduce prison sentence^1,2^  Protection against evil spirits^1,3,6^  Improve physical appearance^3^  Resolve court cases^3,4^  Cold/flu treatment^3^  Improved cognitive ability^7,8^ |
| Mammalia | Artiodactyla | *Capra hircus* | Domestic goat | Entire carcass^1^ | Spiritual offerings^1^  Spiritual rituals^1^ |
| Mammalia | Carnivora | *Caracal caracal* | Caracal | Skin piece^1^  Subcutaneous fat^2^  Ears^3^  Heart^4^  Liver^5^  Bone(s)^6^  Oil^7^ | Remove evil spirits from home^1^  Clothing/decoration^1^  Cold/Flu treatment^1,6^  Protection against evil spirits^2,7^  Predict future^3^  Create conflict^4^  Epilepsy treatment^5^ |
| Mammalia | Primates | *Chlorocebus pygerythrus* | Vervet monkey | Oil^1^  Skin piece^2^ | Protection against evil spirits^1,2^ |
| Mammalia | Artiodactyla | *Connochaetes taurinus* | Blue wildebeest | Skin piece^1^  Fur^2^ | Clothing/decoration^1^  Epilepsy treatment^1^  Remove evil spirits from home^2^ |
| Mammalia | Cetacea | *-* | Dolphin spp. | Bone(s) | Attract good fortune  Improved fertility  Increase livestock numbers  Alcoholism cure  Shingles treatment  Love charm |
| Mammalia | Perissodactyla | *Equus* spp. | Equine spp. | Hoof | Sports betting/gambling |
| Mammalia | Perissodactyla | *Equus caballus* | Domestic horse | Afterbirth | Improved fertility |
| Mammalia | Perissodactyla | *Equus zebra zebra* | Cape mountain zebra | Subcutaneous fat^1^  Entire skin^2^ | Prevent lightning strikes^1^  Improved fertility^1^  Clothing/decoration^2^ |
| Mammalia | Carnivora | *Felis catus* | Domestic cats | Entire carcass | Protect home from evil spirits |
| Mammalia | Carnivora | *Felis libyca* | African wild cat | Subcutaneous fat^1^  Scat^2^  Skin piece^3^  Entire skin^4^  Paws^5^  Head^6^ | Stroke treatment^1,2^  Rituals^3,5,6^  Cause a relationship break-up^3,5,6^  Clothing/decoration^4^ |
| Mammalia | Carnivora | *Galerella pulverulenta* | Cape grey mongoose | Skin piece | Cause misfortune |
| Mammalia | Carnivora | *Genetta* spp. | Genet spp. | Skin piece^1^  Entire carcass^2^  Oil^3^ | Clothing/decoration^1^  Community status^1^  Good luck^1^  Protection against evil spirits^2,3^ |
| Mammalia | Carnivora | *Herpestes ichneumon* | Egyptian mongoose | Skin piece | Protection against evil spirits |
| Mammalia | Artiodactyla | *Hippopotamus amphibius* | Common hippopotamus | Subcutaneous fat | Love charm |
| Mammalia | Carnivora | *Hyaena brunnea* | Brown hyena | Skin piece^1^  Subcutaneous fat^2^ | Clothing/decoration^1^  Attract good fortune^2^ |
| Mammalia | Rodentia | *Hystrix africaeaustralis* | Cape porcupine | Quills^1^  Oil^2^  Muscle tissue^3^  Viscera^4^  Bone(s)^5^  Scat^6^  Entire carcass^7^  Skin piece^8^  Stomach contents^9^  Head^10^ | Stir medicine^1^  Headache treatment^1,4^  Protection against evil spirits^1,2,4,5^  Remove ibekelo^1^  Acupuncture^1^  Rid bad blood^1^  Remove idliso^1,6^  Protect infants from evil spirits^1,10^  Attract good fortune^3^  Epilepsy treatment^4^  Arthritis treatment^4^  Predict future^4^  Reduce prison sentence^4,6^  Repel tokoloshe^6^  Protection against bad muti^7,8^  Stroke treatment^9^ |
| Mammalia | Carnivora | *Ictonyx striatus* | Striped polecat | Skin piece | Create conflict  Protect from being attacked  Headache treatment |
| Mammalia | Lagomorpha | *Lepus* spp. | Hare spp. | Urine^1^  Muscle tissue^2^  Head^3^  Foot^4^  Viscera^5^  Entire carcass^6^ | Return idliso^1^  Improve physical appearance^2^  Epilepsy treatment^3,4,5,6^  Kidney stones treatment^6^  Shingles treatment^6^ |
| Mammalia | Proboscidea | *Loxodonta africana* | African elephant | Oil | Protection against evil spirits |
| Mammalia | Carnivora | *Mellivora capensis* | Honey badger | Skin piece^1^  Paws^2^  Bone(s)^3^  Subcutaneous fat^4^  Oil^5^ | Protection against evil spirits^1^  Protection against bad muti^1,2,3^  Protect infants from evil spirits^4,5^ |
| Mammalia | Artiodactyla | *Oreotragus oreotragus* | Klipspringer | Skin piece | Clothing/decoration |
| Mammalia | Tubulidentata | *Orycteropus afer* | Aardvark | Skin piece^1^  Bone(s)^2^  Oil^3^ | Protection against bad muti^1,2^  Remove evil spirits from home^1,3^  Protection against evil spirits^2,3^ |
| Mammalia | Carnivora | *Otocyon megalotis* | Bat-eared fox | Brain^1^  Urine^2^  Skin piece^3^  Entire skin^4^ | Improved cognitive ability^1,2^  Remove evil spirits from home^3^  Clothing/decoration^4^ |
| Mammalia | Carnivora | *Panthera leo* | Lion | Liver^1^  Bone(s)^2^  Skin piece^3^ | Poison others (idliso)^1^  Paralysis treatment^2^  Clothing/decoration^3^ |
| Mammalia | Carnivora | *Panthera pardus* | Leopard | Skin piece^1^  Oil^2^  Entire skin^3^  Subcutaneous fat^4^  Brain^5^  Vertebrae^6^  Nails^7^  Eye^8^  Bone(s)^9^  Entire carcass^10^  Muscle tissue^11^ | Clothing/decoration^1,3^  Community status^1^  Protection against evil spirits^1,2,4,9^  Mental illness treatment^1,2,5,6,11^  Cold/flu treatment^1^  Epilepsy treatment^2,4^  Increased business success^2^  Attract good fortune^2^  Good luck^2^  Kidney stones treatment^2^  Shingles treatment^4^  Arthritis treatment^7^  Predict future^8^  Jewellery^9^  Headache treatment^11^ |
| Mammalia | Carnivora | *Panthera tigris* | Tiger | Skin piece | *NIA* |
| Mammalia | Primates | *Papio ursinus* | Chacma baboon | Subcutaneous fat^1^  Paws^2^  Skin piece^3^  Head^4^  Palm skin^5^  Oil^6^  Entire carcass^7^  Ash^8^  Bone(s)^9^  Testes^10^ | Acquire wealth^1,2,9^  Protection against bullets^1^  Fertilize crop fields^1,2,6^  Protection against evil spirits^1,2,3,4,6,8,9,10^  Attract good fortune^1,2,3,10^  Black magic^2,5^  Community status^3^  Protect livestock from evil spirits^3^  Clothing/decoration^3^  Remove idliso^3^  Poison others (idliso)^3^ |
| Mammalia | Artiodactyla | *Pelea capreolus* | Grey rhebok | Skin piece^1^  Horn(s)^2^ | Clothing/decoration^1,2^  Used as casing for powders^2^ |
| Mammalia | Artiodactyla | *Phacochoerus africanus* | Common warthog | Skin piece | Clothing/decoration |
| Mammalia | Carnivora | *Poecilogale albinucha* | African striped weasel | Skin piece^1^  Paws^2^  Entire carcass^3^ | Protect livestock from evil spirits^1^  Unify livestock^2^  Protection against evil spirits^3^ |
| Mammalia | Artiodactyla | *Potamochoerus larvatus* | Bushpig | Skin piece | Protection against evil spirits |
| Mammalia | Hyracoidea | *Procavia capensis* | Rock hyrax | Paws^1^  Viscera^2^  Skin piece^3^  Fur^4^ | Promote physical growth^1^  Fertility treatment^2^  Increase livestock fertility^3,4^  Manipulate sex of stock offspring^3^ |
| Mammalia | Carnivora | *Proteles cristata* | Aardwolf | Entire skin^1^  Skin piece^2^  ^3^Bone(s) | Clothing/decoration^1^  Attract good fortune^2,3^ |
| Mammalia | Artiodactyla | *Raphicerus melanotis* | Cape grysbok | Skin piece | Clothing/decoration  Prevents road accidents |
| Mammalia | Perissodactyla | *Ceratotherium/*  *Diceros sp.* | Rhinoceros spp. | Horn | Attract good fortune |
| Mammalia | Artiodactyla | *Sus scrofa* | Feral pig | Bone(s) | Arthritis treatment |
| Mammalia | Artiodactyla | *Sylvicapra grimmia* | Common duiker | Skull^1^  Skin piece^2^  Horn(s)^3^  Hoof^4^ | Invite spiritual presence (rituals)^1^  Clothing/decoration^2^  Jewellery^3^  Protection against evil spirits^4^ |
| Mammalia | Artiodactyla | *Syncerus caffer* | African buffalo | Subcutaneous fat^1^  Bone(s)^2^  Horn(s)^3^ | Protect home against evil spirits^1,3^  Protection against evil spirits^1^  Paralysis treatment^2^  Exorcism^3^ |
| Mammalia | Artiodactyla | *Taurotragus oryx* | Common eland | Horn(s) | Exorcism |
| Mammalia | Artiodactyla | *Taurotragus* spp. *& Tragelaphus* spp. | Spiral-horned antelope spp. | Horn(s) | Protect home against evil spirits  Exorcism |
| Mammalia | Artiodactyla | *Tragelaphus angasii* | Nyala | Horn(s) | Exorcism |
| Mammalia | Artiodactyla | *Tragelaphus strepsiceros* | Greater kudu | Horn(s) | Exorcism  Clothing/decoration |
| Mammalia | Artiodactyla | *Tragelaphus sylvaticus* | Cape bushbuck | Skull^1^  Horn(s) | Invite spiritual presence (rituals)^1^  Exorcism^2^ |
| Mammalia | Carnivora | *Vulpes chama* | Cape fox | Entire skin^1^  Brain^2^  Urine^3^ | Clothing/decoration^1^  Improved cognitive ability^2,3^  Headache treatment^3^ |
| Reptilia | Squamata | *Agama atra* | Southern rock agama | Entire carcass^1^  Entire skin^2^ | Protection against evil spirits^1,2^ |
| Reptilia | Squamata | *Bitis arietans* | Puff adder | Bone(s)^1^  Oil^2^  Skin piece^3^  Head^4^  Tail^5^  Entire carcass^6^ | Exorcism^1^  Protection against evil spirits^2,3,4,5^  Arthritis treatment^3^  Protect livestock from evil spirits^6^  Send evil spirits to kill livestock^6^  Protect home from evil spirits^6^  Remove evil spirits from home^6^  Remove ibekelo^6^ |
| Reptilia | Testudines | *Chersina angulata* | Angulate tortoise | Blood^1^  Entire carcass^2^  Carapace^3^ | Epilepsy treatment^1^  Protection against evil spirits^2^ |
| Reptilia | Squamata | *Cordylus* spp. | Girdled lizard spp. | Entire carcass | *NIA* |
| Reptilia | Crocodilia | *Crocodylus niloticus* | Nile crocodile | Bone(s)^1^  Vertebrae^2^  Subcutaneous fat^3^ | Protect home from evil spirits^1,2^  Protection against evil spirits^3^ |
| Reptilia | Squamata | *Dendroaspis* spp. | Mamba spp. | Skin piece^1^  Bone(s)^2^  Subcutaneous fat^3^  Entire carcass^4^ | Protection against evil spirits^1,2^  Remove evil spirits from home^1,3^  Exorcism^4^ |
| Reptilia | Squamata | *Hemachatus haemachatus* | Rinkhals | Entire carcass^1^  Ash^2^ | Protect crops from hail damage^1^  Protection against evil spirits^2^ |
| Reptilia | Squamata | *Naja nivea* | Cape cobra | Skin piece^1^  Head^2^  Tail^3^ | Remove evil spirits from home^1^  Protection against evil spirits^1,2,3^ |
| Reptilia | Squamata | *Pseudaspis cana* | Mole snake | Skin piece | Protection against evil spirits |
| Reptilia | Squamata | *Python sebae* | African rock python | Skin piece^1^  Bone(s)^2^  Entire carcass^3^ | Protection from ibekelo^1^  Protection against evil spirits^1^  Remove evil spirits from home^1^  Remove ibekelo^2^  Exorcism^3^ |
| Reptilia | Squamata | *-* | Snake spp. | Entire carcass | Protection against evil spirits |
| Reptilia | Testudines | *Stigmochelys pardalis* | Leopard tortoise | Scat | Epilepsy treatment |
| Reptilia | Squamata | *Varanus genus* | Monitor lizard spp. | Muscle tissue^1^  Skin piece^2^  Paws^3^  Oil^4^ | Stop bad dreams from occurring^1^  Idliso treatment^1^  Protection against evil spirits^1,2,3^  Rid bad luck^2,4^  Prevent a miscarriage^2^  Arthritis treatment^2^  Kidney stones treatment^2^ |
